# Tecido Adiposo Epicárdico: Um Novo Parâmetro de Imagem Cardiovascular Profundamente Relacionado às Doenças Cardiovasculares

**DOI:** 10.36660/abc.20200309

**Published:** 2020-11-01

**Affiliations:** 1 Hospital Universitário Antônio Pedro NiteróiRJ Brasil Hospital Universitário Antônio Pedro, Niterói, RJ - Brasil

**Keywords:** Pericárdio, Tecido Adiposo, Obesidade, Criança, Resistência à Insulina, Ecocardiografia/métodos

Prezado Editor,

Lemos com grande interesse o artigo intitulado “A relação entre o tecido adiposo epicárdico e a resistência à insulina em crianças obesas”.^1^ Nesse artigo, os autores realizaram ecocardiografia doppler tecidual para avaliar a espessura do tecido adiposo epicárdico (TAE), retardo eletromecânico (REM) e outras medições padrão em 94 pacientes pediátricos obesos.^1^ Pacientes com Avaliação do Modelo Homeostático para Resistência à Insulina (HOMA-IR) maior que o percentil 90 em uma curva de percentil específica para idade e sexo foram rotulados como tendo resistência à insulina (RI).^1^ O estudo apontou que o grupo com RI tinha espessura significativamente maior de TAE do que os pacientes incluídos sem RI (7,15 vs. 5,5 mm, p<0,004, respectivamente) e REM inter e intra-atrial prolongado no grupo IR em comparação com o grupo não IR (p<0,010; p=0,032, respectivamente).^1^ Além disso, os autores verificaram que um valor de corte para TAE de >3,85 mm poderia predizer RI com 92,5% de especificidade e 68,5% de sensibilidade (p=0,002).^1^ O estudo concluiu que o TAE poderia ser usado para identificar RI em crianças, pois afirma-se que o TAE é parâmetro barato e acessível que pode ser facilmente medido com ecocardiografia.^1^

Em relação ao REM, associado ao desenvolvimento de fibrilação atrial (FA),^1^ seria interessante realizar uma análise multivariada para determinar se a espessura do TAE está independentemente associada ao REM. O TAE é um depósito de gordura visceral localizado ao redor do miocárdio e do pericárdio, secretando diversas quimiocinas e citocinas pró-inflamatórias, coletivamente denominadas de adipocinas.^2^ Devido à proximidade do TAE com o miocárdio, a gordura epicárdica pode promover efeitos locais inflamatórios e mecânicos no músculo cardíaco e nos vasos coronarianos. O TAE também exerce um papel cardioprotetor no coração, prevenindo os efeitos tóxicos dos ácidos graxos livres de alta circulação no miocárdio e nas artérias coronárias, reduzindo a tensão vascular e prevenindo a remodelação vascular.^2^ Estudos anteriores também observaram uma associação independente entre TAE e doenças cardiovasculares como a doença arterial coronariana, insuficiência cardíaca e FA.^2^ Uma metanálise de Gaeta et al.,^3^ mostrou uma associação entre TAE e FA.^3^ Portanto, o TAE poderia ter um papel na gênese da arritmia, pois poderia ser um modulador, substrato ou gatilho no desenvolvimento de FA^3^ e seria interessante investigar sua associação com o REM.

No entanto, embora os achados desse estudo^1^ tenham sido interessantes, devemos levar em consideração a sensibilidade consideravelmente baixa da mensuração do TAE para identificar RI em pacientes pediátricos obesos (68,5%). Os testes de triagem devem ter alta sensibilidade para minimizar os riscos de falsos negativos. Além disso, embora a ecocardiografia possa ser mais barata do que certos exames laboratoriais em alguns países, como o Brasil, a ecocardiografia é mais cara na maioria dos países por ter alto custo de mão de obra humana associado. Além disso, o TAE não costuma ser medido em laboratórios de ecocardiografia, nem a ecocardiografia é o método padrão ouro para medir o TAE, conforme citado pelos autores.
