# Supplementary material for: Tecido Adiposo Epicárdico: Um Novo Parâmetro de Imagem Cardiovascular Profundamente Relacionado às Doenças Cardiovasculares
Source: Arq Bras Cardiol. 2020 Nov 1;115(5):971–2. [Article in Portuguese] doi: 10.36660/abc.20200309 (PMC8452219; doi:10.36660/abc.20200309)
Supplement: Translation

# Translation: Epicardial Adipose Tissue: A New Cardiovascular Imaging Parameter Deeply Connected with Cardiovascular Diseases

**Affiliations:** 1 NiteróiRJ Brazil Hospital Universitário Antônio Pedro, Niterói, RJ - Brazil

**Keywords:** Pericardium, Adipose Tissue, Obesity, Child, Insulin Resistance, Echocardiography/methods

Dear Editor,

We read with great interest the article entitled “The Relationship Between Epicardial Adipose Tissue and Insulin Resistance in Obese Children”.^1^ In that paper, the authors performed tissue doppler echocardiography to evaluate epicardial adipose tissue (EAT) thickness, electromechanical delay (EMD) and other standard measurements in 94 obese pediatric patients.^1^ Patients with Homeostatic Model Assessment for Insulin Resistance (HOMA-IR) greater than the 90^th^ percentile in an age and sex-specific percentile curve were labeled as having insulin resistance (IR).^1^ This study found that the group with IR had significant higher thickness of EAT than patients included without IR (7.15 vs. 5.5 mm, p<0.004, respectively) and inter and intra-atrial EMD was prolonged in the IR group compared to the non-IR group (p<0.010; p=0.032, respectively).^1^ Moreover, the authors found that a cut-off value for EAT of >3.85 mm could predict IR with 92.5% specificity and 68.5% sensitivity (p=0.002).^1^ The study then concluded that EAT could be used to identify IR among children, as it is stated that EAT is a cheap and accessible parameter that could be easily measured with echocardiography.^1^

Regarding EMD, which is associated with atrial fibrillation (AF) development,^1^ it would be interesting to perform a multivariate analysis to determine whether EAT thickness is independently associated with EMD. EAT is a visceral fat store located around the myocardium and pericardium that secretes several proinflammatory chemokines and cytokines, collectively called adipokines.^2^ Due to the proximity of EAT with the myocardium, epicardial fat may promote local inflammatory and mechanical effects on the cardiac muscle and coronary vessels. EAT also has a cardioprotective role on the heart by preventing the toxic effects of high-circulating free fatty acids on the myocardium and coronary arteries, lowering vascular tension and preventing vascular remodeling.^2^ Previous studies have also observed an independent association between EAT and several cardiovascular diseases such as coronary artery disease, heart failure and AF.^2^ A meta-analysis by Gaeta et al.^3^ showed an association between EAT and AF.^3^ Therefore, EAT could have a role in arrhythmia genesis as it could be a modulator, substrate or trigger in AF development^3^ and it would be interesting to investigate its association with EMD.

However, although the findings of this study^1^ were interesting, we should take into account the considerably low sensitivity of measuring EAT to identify IR in obese pediatric patients (68.5%). Screening tests should have high sensitivity to minimize the risks of false negatives. Moreover, although echocardiography might be less expensive than certain laboratory tests in some countries, such as Brazil, echocardiography is more costly in the majority of countries as it has a high human labor cost associated. In addition to that, EAT is not usually measured in echocardiography laboratories, neither echocardiography is the gold standard method to measure EAT, as mentioned by the authors.
